# Dr. Kinglake, on Influenza

**Published:** 1803-06-01

**Authors:** Robert Kinglake

**Affiliations:** Taunton


### *To the Editors of the Medical and Phyſical Journal.*

GENTLEMEN,

YOUR call for contributing any practical information, which promises to be extensively useful by publication, on the prevailing catarrhal affection, under the name of *Influenza,*

*Influenza*, is rendered truly imperious, by the ravage it is still making in certain circumstances.

It may be more curious than instructive to enter into a lengthened disquisition on the origin of this epidemic malady, but a brief inquiry into its probable source will most likely best illustrate the mode of treatment which, when early resorted to, has proved under my direction invariably successful. It is not difficult to admit, that the very variable course of atmospheric temperature, which has distinguished the weather during the four preceding months, must as usual have impeded, in weakly and irritable temperaments, an equal and salutary distribution of the circulating fluids. It is also no less obvious, that a surcharge on the lungs, more particularly on the bronchial glands, would be more quickly announced by the characteristic symptoms of coughing, difficulty of breathing, and the other common criteria of catarrhal ailment, than could occur if the redundancy should alight on any other organ. This pulmonic precedency in morbid influence, from diminished perspiration and consequent vascular tumult, serves at once to induce and further invite diseased determination to the lungs. The continuance of this affection, whether from its original violence or mis-management, associatively excites diseased actions throughout the system. The hepatic organ occasionally sympathises to the extent of burthening the first passages with an increased quantity of its bilious secretion. When the more pure catarrhal aspect of the complaint has been disfigured by this morbid redundancy of bile, a yellow hue has both pervaded the cuticular surface, and tinged the various secreted fluids. This icteric accompaniment imparts to the disorder an unsightly mien, which, in connection with the prevailing febrile agitation, extreme debility, and mental depression, may impose itself on either the timid or inexperienced, as allied to the memorable yellow fever, which lately, by its desolating fatality, so deplorably thinned the population of America. Whatever disguises may happen, either from temperamental or accidental causes, the *influenzal* epidemic of the present period, in no instance, loses either its catarrhal form or nature. It tenaciously retains its specific character throughout its progress; nor even in the shades of death does it exhibit a typhoid feature. It is unquestionably contagious, so are common catarrhs; in this respect it differs only in the *majus* and *minus*. The existing *influenza* induces these changes in the salutary state of pulmonary action that arrange, constitute, stitute, and evolve a peculiar halitus, which reaching the tracheal membrane, impresses its excitability, and generates an action, as specific of its own nature, as could be respectively produced by variolous, vaccine, venereal, morbillous, or any other description of virus."

The contagious power of this malady is in general traceable by personal intercourse. It may indeed individually originate in the physical circumstances which were before observed to have given it birth; but the prevailing unimpressibillity of the human temperament, by the operation of general causes, requires for its propagation, the concentrated power it obtains from organic preparation. It is no objection to the contagious nature of this disease, that it is not universally operative. Temperamental insusceptibility to be impressed by morbid powers, occasionally resists the utmost force that could be exerted short of positive disorganization.

This counter-acting inaptitude is not unfrequently manifested in the most indubitably infectious diseases incident to human nature.

Morbid actions, of whatever description, generate and evolve a certain power, whether material or motive, capable of inducing a similar action in apt circumstances of temperamental susceptibility; but happily for mankind, this necessary fitness to give effect to contagious agency, does not often exist, and perhaps the capability of originating as well as the liability of taking diseases may be more justly imputed to the errors of social than of natural life.

The catarrhal form of the *influenzal* disease, implies that it is of an inflammatory nature. The severe local irritation which often obtains in different parts of the lungs, confirms it to be of that description.

The intention of cure then, must be exclusively directed to reducing the inflammatory action more or less violently stationed on the bronchial membrane, and sympathetically extended to the system at large; reflection obviously suggests, what experience has abundantly proved, that this indication is most effectually fulfilled, by observing a uniformly *cooling*, not *debilitating** plan of treatment.

The

* It is an erroneous notion that occasional refrigeration and abstinence in disease, weaken more than a heating and stimulating treatment. The native energy of healthy power is certainly reduced both by the abstraction

The patient should abstain from every kind of stimulant, whether dietetic or medicinal; the apartment should be without fire, and incessantly ventilated, occasionally from the window, and constantly from the door; or if strength will permit, confinement in bed should be avoided, and atmospheric exposure, in shady situations, should be preferred. Small quantities of cold water, or toast and water, not exceeding a wine-glass full at a time, should be drank every ten minutes.

If recourse be had to this treatment in the incipient stage of the disease, the complaint will not differ from the slightest attack of catarrh; the local pain will soon cease, and with that, the systematic irritation, dyspnœa, cough, and expectoration. On the contrary, if the plan should be delayed, (as it has in several instances where my advice has been desired) the disease will have suffered much unnecessary aggravation, the patient will breathe tightly, often laboriously, the pulse will become rapid, small, and hard, cough dry and urgent, skin hot and parched, thirst insatiable, local pain unremitting, countenance yellow, debility extreme, nausea incessant, and mental depression distressingly irksome.

Under these circumstances it is highly probable that the disease has been thought, if not originally typhoid, to have now evidently become so. The treatment which would be appropriate to this opinion, according to the prevailing (but in my judgment, erroneous notion of putrid disorders) would so insure, and precipitate the dissolution of the patient, as to preclude all calculable chance of escape.

My experience authorizes me to say, that the benefit of abstracting heat, by atmospheric exposure, light bed-clothes, copious dilution with cold water, and avoiding stimulants of every description, will almost certainly rescue the patient from danger, and leave nothing more for medicine to do, than gently to move the bowels in case of costiveness, and at most, to aid in the refrigerating plan, by the milder sudorifics, such as the aqua ammonia acetata, spiritus ætheris nitrosi, spiritus ætheris vitriolici, vinum antimonii tartarisati, &c. The custom of diluting with steaming fluids, though slender in quality, is hurtful. They do not transfer redundant heat like cold liquids.

tion and increase of excitement, but by its due diminution vital force may be said to be nursed, while undue stimulant agency tends to dissipate it even to extinction; hence a moderate negation of excitement debilitates much less *directly*, than its excessive employ does *indirectly*.

The

The salutary influence of this treatment is evinced by perspiration soon following a course of dilution with cold water. The morbidly contracted state of the cuticular exhalents, from the excitement of disease, is increased, not diminished by either stimulating or warm fluids. This diseased condition, so obstructive to salutary perspiration, is naturally and quickly overcome by diluting with cold liquids, and atmospheric exposure.

The local pain may be subdued by the application of a blister over the aggrieved part, or when not urgently violent, more commodiously by a stimulant ointment, composed of antim. tartarisat. dram. j. adip. suillæ unc j. gradatim conterantur. The bulk of a nutmeg of this ointment may be rubbed on the chest, or over the affected part, every eight hours, until pustular eruptions be produced, which usually follow within twenty-four hours. It may be expedient to support this eruption for some weeks, or at least during the period of convalescence, to obviate a relapse of morbid determination to the lungs. Indeed, the counter stimulus induced by the continued topical application of this ointment, unremittedly acts as a salutary derivant from the morbidly irritable membrane investing the trachea, and its bronchial tubes, thereby tending to prevent an habitual establishment of the diseased action, which, though yielding in its more acute form, may entail a lingering duration, ultimately productive of tubercular lesion, and consequent pulmonary consumption. Bleeding is not indicated in this disease, nor is brisk intestinal evacuation. The debility which ensues either of these attempts to relieve, is not compensated by a proportionate diminution of the local pain, while the reduction of systematic energy farther deranges the natural distribution of the vascular fluids, loads the diseased parts, and either induces a necessity for tedious expectoration, or occasions a suffocating degree of serous effusion in the bronchia.

The debility, whether direct or indirect, induced by excessive evacuation in the former case, and by stimulant means of cure in the latter, has in most of the fatal instances in my knowledge, probably occasioned an irremediable effusion in the bronchial tubes; irremediable, because the existing arterial atony has been incapable of urging the effused fluid into the lymphatic course of serous circulation.

It is always inadmissible to reduce systematic energy, either by inordinate evacuations or excessive excitement, when the native conditions of health may be efficaciously restored by repressing the morbidly stimulant influence of heat. An unremitted course of suitable refrigeration keeps the *Influenza* of the present season within safe limits, resists the evolution of threatening symptoms, and ensures it a healthful termination.

An importunate sense of duty irresistibly prompts me to request, that you will insert in the next number of your Journal, this hasty sketch of my opinion and practice, relative to the prevailing Epidemic, with a view to suggest to the medical world, that the disease does not appear to differ in any essential circumstance from common catarrh; that its disguised, and worst forms, as well as its fatal termination, seem to have arisen more from a want of timely, uniformly, and perseveringly reducing the prevailing temperature, by a rigidly cooling plan of treatment, than from its malignant or mortal nature; and finally, (what is not sufficiently adverted to) that the early institution of this mode of remedy, most advantageously obviates that extreme degree of indirect debility, which a neglected or delayed observance of it occasions, by permiting the exhausting career of morbid conflict to proceed unchecked, by which the period of convalescence is vastly protracted, and glandular consumption, with other fatal forms of chronic affection, imminently endangered.

*Taunton,* I am, &c.

*April* 27, 1803. ROBERT KINGLAKE.

